# Validation of the Arabic version of medication adherence report scale questionnaire and beliefs about medication -specific questionnaire: A factor analysis study

**DOI:** 10.1371/journal.pone.0266606

**Published:** 2022-04-12

**Authors:** Walid Al-Qerem, Abdel Qader Al Bawab, Osama Abusara, Nimer Alkhatib, Robert Horne

**Affiliations:** 1 Department of Pharmacy, Faculty of Pharmacy, Al-Zaytoonah University of Jordan, Amman, Jordan; 2 Al-Zaytoonah Health Technology and Innovation Office, Al-Zaytoonah University of Jordan, Amman, Jordan; 3 Centre for Behavioral Medicine, UCL School of Pharmacy, University College London, London, United Kingdom; University of Turin, ITALY

## Abstract

Medication Adherence Report Scale questionnaire (MARS-5) and the Beliefs about Medication Questionnaire-specific (BMQ-specific) are well known tools to assess adherence to medication and beliefs of chronic patients. However, validated Arabic versions of such questionnaires are lacking. We aim to validate the Arabic versions of BMQ-specific and MARS-5. In this study, a cross-sectional study was performed between November 2019 and March 2020. Participants were reached from secondary and tertiary care clinics in Jordan. Exploratory factor analysis (EFA) and Confirmatory Factor analysis (CFA) were conducted to validate the employed questionnaires on the tested sample. The internal consistency of the questionnaires was assessed by calculating Cronbach’s alpha, and Cronbach’s alpha if item is deleted. A total of 485 patients who met the inclusion criteria were recruited. The mean age of the participants was 57.14 years (22–82 years); and 39% of the participants were older than 65 years. The most common chronic diseases reported by participants were hypertension and diabetes mellitus, 35.7 and 32.2% respectively. EFA suggested two-factor model for BMQ-specific and one-factor model for MARS-5 which was confirmed by CFA analyses. The resulted Cronbach’s alphas of the questionnaires ranged from 0.89–0.93. Both analyses showed that the Arabic versions of both MARS-5 and BMQ-specific are valid and can be used for the suggested study population. Further validation-based research may enhance the transcultural adaptation of such questionnaires.

## Introduction

Chronic diseases are predominant among adult and geriatric populations, which may necessitate the need of multi drug use “polypharmacy” [1]. Elderly patients’ polypharmacy influences their attitudes towards complying with therapeutic regimens and may lead to non-adherence [2, 3]. Non-adherence to medications is a major health problem that is associated with treatment failure.

In Jordan, non-communicable chronic diseases are major cause of deaths. In 2005, the prevalence of non-communicable chronic diseases ranged from 7.5%-14.8%. The number is expected to increase in 2050 to approximately 28.9%-37.3%. In 2015, the top death-related chronic diseases in Jordan were ischemic heart disease (prevalence 6.4%), cerebrovascular disease (prevalence 14.8%), and diabetes (prevalence7.5%) [4, 5]. Non-communicable chronic diseases accounted for 78% of deaths in 2018 [6]. Several published studies have reported low adherence in Jordanian patients with several chronic diseases including diabetes mellitus and dyslipidemia [7–9], which influenced health outcomes [10]. Similar to the other parts of the globe, the issue of non- adherence to medications in Jordanian chronically ill patients is challenging. Reported prevalence of non-adherence to medications ranged from 29.6%-64.2% in different clinical settings [7, 8, 11, 12]. More specifically, adherence to medications in diseases like hypertension, dyslipidemia and diabetes mellitus were reported to be 81%, 73.2% and 58.7%, respectively [8, 9, 13]. Studies showed that low level of adherence is associated with several factors, such as patient, disease, and medication-related factors [14]. The use of medication among patients is also affected by their perception [15]. Questionnaires were developed to explore the association between patients’ beliefs and their adherence towards medications. “Beliefs about Medications Questionnaire-(general or specific) (BMQ-general/specific)” and “Medication Adherence Report Scale-(10 or 5) (MARS-10/5)” are two valid and reliable questionnaires developed by Horne *et al* [16–21]. BMQ assess positive and negative feelings by measuring essential feelings and fears from medications. MARS-10 is a scale of 10 items that assesses intentional (“I avoid using it if I can”) and nonintentional nonadherence (“I forget to use it”), while MARS-5 is a shorter version of MARS-10 that is modified to offer more details and differentiability between individuals [19].

Transcultural adaptation of BMQ or MARS questionnaires should be performed for the Jordanian population. Although, BMQ and MARS-5 were previously translated to Arabic, nevertheless, there were lacking statistical analysis, or at least the validation was conducted in different care settings than those suggested in this study. For example Alhalaiqa et al (2015) used Pearson Product Moment Correlation Coefficient to test-retest the reliability of the questionnaire and Cronbach’s alpha to evaluate internal consistency [22] but did not conduct factor analysis, while Alsous et al (2017) concluded the validity and reliability [23] on Jordanian children with chronic diseases and their parents, and didn’t include adult Jordanians with chronic diseases [22]. Moreover, Alsous et al. did not conduct confirmatory factor analysis (CFA). Therefore, to the best of our knowledge this is the first confirmatory factor analysis study that validated BMQ and MARS-5 on adult Jordanian patients.

The aim of this study is to validate Arabic-translated questionnaires of BMQ-specific and MARS-5 used to measure adherence to chronic medications in Jordanian adult patients in relation to patients’ beliefs about medications and sociodemographic characteristics. Exploratory and confirmatory factor analyses were performed to confirm the validity status of these questionnaires.

## Materials and methods

### Study design and participants

The study design is compliant with the ethical standards of Declaration of Helsinki guidelines that are stated by the World Medical Association. The Institutional Review Board (IRB) of Al-Zaytoonah University of Jordan approved the study protocol (approval reference number: 07.02.2019). A signed Informed Consent Form (ICF) was obtained from the participant who agreed to take part in this study. Every participant provided a signed (written) ICF after reading the study information sheet. Confidentiality of all the participants was well maintained throughout the study.

This is a cross-sectional study that was carried out November 2019 and March 2020, and involved 485 patients who are diagnosed with one or more chronic diseases and take prescribed medications for at least one year (as per CDC definition) and aged 20 years or older. Participants with chronic diseases were recruited from secondary and tertiary care settings clinics across Jordan. The clinics included specialties like cardiovascular, respiratory, internal medicine and endocrinology. Chronic disease diagnosis was confirmed from patient files. Patients who were severely ill and cannot respond, and patients who were less than 20-year-old were not included in the study.

### Data collection

The first part of the questionnaire consisted of queries to collect socio-demographic data about the participants. These included age, gender, income level, educational level, diagnosis, duration of illness, the number of medications, type of medications, and insurance status. Medications prices were estimated as per local prices in Jordanian Dinar (JOD). Income levels were categorized in terms of household income to low (less than 500 JOD), intermediate (between 501 and 1000 JOD) and high (more than 1000 JOD) based on average household monthly income in Jordan [24].

A self-reported questionnaire “Medication Adherence Report Scale (MARS-5)” was included in the second part of the questionnaire and used to assess the participants’ level of adherence toward their prescribed medications. The MARS-5 has been widely used in studies on a variety of chronic illnesses, including type two diabetes, hypertension, and chronic obstructive pulmonary disease [23]. The MARS-5 is composed of five questions about “forgetting”, “changing of dosages”, “stopping”, “skipping” and “using medication less than what is prescribed”. Study subjects indicated the frequency as (“always”, “often”, “sometimes”, “rarely” or “never”) for each question, with ascending scores from “always” (1 point) to “never” (5 points). Scores for each of the five questions are aggregated to give the final score, which ranges from 5 to 25 points. The cutoff point of adherence in the current work was determined to be ≥80% of the aggregated MARS-5 scores for the target population (i.e. MARS-5 score ≥20).

The third part included a self-reported questionnaire; the Beliefs about Medicines Questionnaire (BMQ-specific) [16]. This well-validated tool is used to measure patients’ perceptions and insights of a particular medication in more definite situations such as chronic illnesses. BMQ-Specific consists of 11-items that incorporates two subscales: The specific-necessity subscale that is assessing the patient’s beliefs about the necessity and need of the prescribed medication, and the specific-concerns subscale, which addresses the patients’ concerns and worries regarding potential adverse outcomes from the medications’ use. Respondents indicate their degree of agreement with each statement about medicines on a five-point Likert scale, in which score 1 represents a strong disagreement and 5 epitomizes a strong agreement. Scores obtained for the individual items within each scale are summed to give a scale score in which the higher scores indicate stronger beliefs in the concepts represented by the scale. Professor Robert Horne and his colleagues developed BMQ, as a method for assessing cognitive representations of medication. It has been confirmed that high concern and low necessity scores have been correlated with high levels of non-adherence in a number of chronic illnesses.

Both MARS-5 and BMQ-specific questionnaires were translated from English language to Arabic language; the translations were validated by the back-translations technique and involved three qualified independent translators. A well-trained research assistant (pharmacist) was employed to administer the questionnaires to the recruited patients who met the inclusion criteria in order to extract their own perceptions in a neutral manner.

### Data analysis

Analysis was performed using the IBM SPSS Statistics software version 27 (Armonk, New York, USA) and AMOS 26. Continuous variables were tested for normality of distribution. Data were presented as frequency (%), mean ± standard deviation. Exploratory factor analysis (EFA) was conducted to validate the Arabic translated versions of MARS-5 and BMQ-specific on the tested sample. To assess the suitability of the data for EFA, Kaiser-Meyer-Olkin value (KMO), and Bartlett’s Sphericity Test, were all applied. Communalities were analyzed and any question with a communality less than 0.35 was removed from the data. Parallel analysis and scree plot were used to determine the number of factors most suitable for study data. Scree plot is a line plot of the eigenvalues of factors in an analysis which is used to determine the number of factors that should be retained in a model [25]. The inflection point in the scree plot line is identified and the number of factors is determined based on the number of eigenvalues present in the plot line above the inflection point [26].

Since the correlations of the produced factors in BMQ were less than the 0.32 cut-off point, orthogonal rotation (varimax rotation) was used. In MARS-5 only one factor was produced, therefor no rotation was needed. To note, a two-factor model indicates that the items in the questionnaire produce two independent latent constructs, while the one-factor model indicates that all the items in the questionnaire are highly correlated and represents one latent construct. Any item that had a loading below 0.4 in all factors, or had a loading of 0.4 or more in multiple factors was excluded. To evaluate the internal consistency of each factor, Cronbach’s alpha, and Cronbach’s alpha if item is deleted were calculated. CFA was applied using maximum likelihood (ML) approach on the suggested final model, goodness of fit was evaluated by calculating CMIN/DF (minimum discrepancy), GFI (goodness of fit index), CFI (comparative fit index), and standardized root mean squared residual (SRMR) and RMSEA (Root Mean Square Error of Approximation). Acceptable values for CMIN/DF are 2–5, for RMSEA are 0.05–0.08 and for AGFI, GFI, and CFI values closer to 1 and for SRMR ≤ 0.05 [27]. The ceiling and floor effects were evaluated by measuring the frequencies of participants who scored the maximum possible or lowest possible scores; in which, the acceptable percentage is less than 15% [28].

Convergent validity and discriminant validity were evaluated in models that had more than one factor by computing Average Variance Extracted (AVE), and Composite Reliability (CR). Acceptable AVE are values above 0.5 [29], and acceptable CR are values above 0.7 [30]. To note, Convergent Validity is confirmed when factor loading are high in the intended factor, and CR and AVE values are acceptable [31]. Discriminant validity can be evaluated by examining items cross-loadings and applying Fornell & Larcker criterion [31] that assumes that the discriminant validity is confirmed when the square root of (AVE) is higher than the correlation between latent constructs [32].

### Sample size calculations

Different techniques were suggested to measure the minimum number of participants required to conduct EFA. These techniques are based either on the total sample number or on item-subject ratio. The first technique suggested different numbers starting from 50 subject [33], and focuses only on the number of participants regardless of the number items “questions” included in the questionnaire, while the later technique focuses on the number of items “questions” when determining the minimum required sample size to conduct factor analysis. This technique recommended different item-subject ratios, however, the highest recommended commonly used item-subject ratio is 1:20 [34]. As the longest questionnaire used in this study contains 11 items, the minimum required sample size is 220.

## Results

### Demographic characteristics of the participants

During the study period, a total of 485 patients (Table 1) met the inclusion criteria and agreed to sign the consent form after reviewing our study overview. The mean age of the participants was 57.1 years (ranged between 22 and 82 years). More than one third of the participants (38.9%) were considered old patients (i.e. > 65 years old). Gender was distributed equally among participants (50.7% were males).

**Table 1:** Demographic characteristics of the participants.

Sample characteristics	Frequency (%) or Mean (SD)
**Gender**	
Male	246 (50.7%)
Female	239 (49.3%)
**Age**	57.1 (12.8)
**Educational level**	
Illiterate	16 (3.3%)
Primary education	55 (11.3%)
Secondary education	49 (10.1%)
High school	130 (26.8%)
Bachelor’s degree	196 (40.4%)
Master’s degree	27(5.6)
PhD	12(2.5)
**Income level**	
Low	57 (11.8%)
Intermediate	374 (77.1%)
High	54 (11.1%)
**Diseases**	
Hypertension	173 (35.7%)
Diabetes	156 (32.2%)
Cardiovascular	88 (18.1%)
Asthma	52 (10.7%)
**Daily frequency of taking medications**	3.5 (1.9)

The majority of participants (77.1%) had intermediate monthly income as per the Jordanian Department of Statistics, and 40% of total participants had completed a bachelor degree. Furthermore, 22% of the total participants had two or more medical conditions. Hypertension was the most prevalent disease among the participants (35.7%) followed by diabetes (32.2%).

### Questionnaires validation

#### Exploratory factor analysis

**Fig 1:**
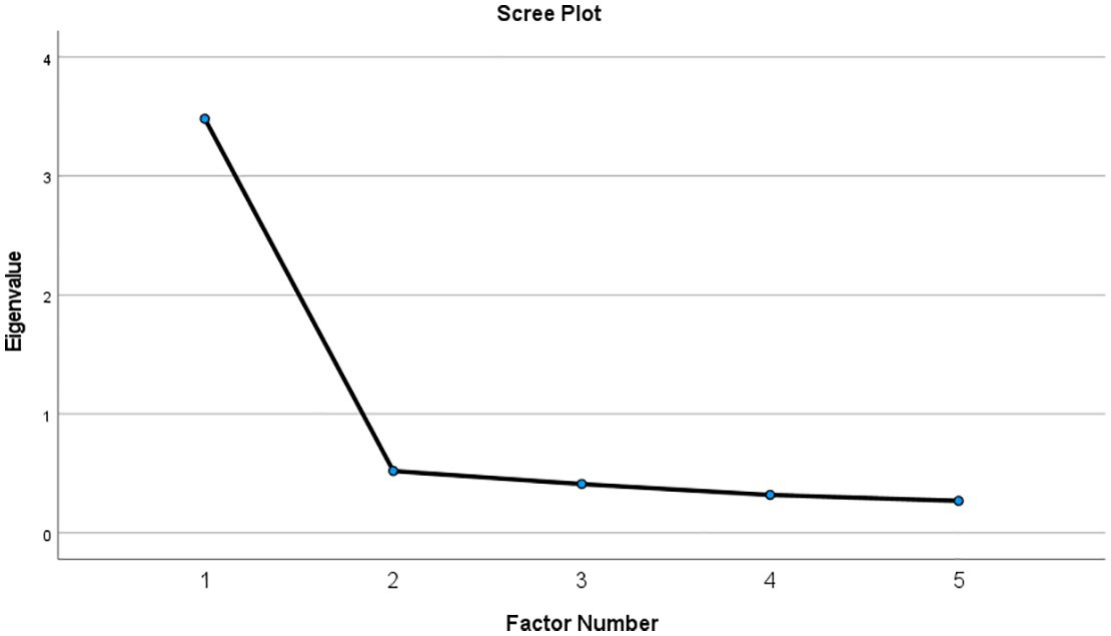
Scree plot of MARS-5.

EFA was conducted to validate the use of MARS-5 among Jordanian adult patients with chronic diseases. KMO and Bartlett’s Sphericity Test confirmed the suitability of the data for factor analysis (0.8, p<0.001). Scree plot (Fig 1) suggested one factor-model as there was only one eigenvalue point above the "elbow" of the graph.

The mean scores of the five items of MARS-5 questionnaire ranged from (3.21–5.51). Internal consistency was confirmed by Cronbach’s alpha (0.8) and corrected item-total correlations. The results also indicated that the reliability was not improved if any item was deleted from models. Internal consistency was deemed adequate if Cronbach’s alpha scores were 0.7 or higher.

The item with highest factor loading and communalities was “I take medications less than instructed”, while the item with the lowest factor loading and communalities was “I forget to take them”. Validation parameter of MARS-5 are presented in Table 2.

**Table 2:** Scores, factor loadings, communalities, corrected item total correlation, and Cronbach’s alpha if item deleted for MARS-5 items.

	Mean score (SD)	Factor loading	Communalities	Corrected Item-Total Correlation	Cronbach’s Alpha if Item Deleted
**I forget to take them**	3.21 (1.10)	0.76	0.59	0.65	0.89
**I change the dose**	3.46 (1.10)	0.81	0.66	0.70	0.87
**I stop taking them for a while**	3.39 (1.13)	0.85	0.72	0.75	0.86
**I decide to skip a dose**	3.51 (1.12)	0.87	0.75	0.78	0.86
**I take medications less than instructed**	3.49 (1.14)	0.88	0.77	0.79	0.85

The overall specific and necessity scores for BMQ-Specific questionnaire were 3.67 and 3.28 respectively. EFA was conducted to validate the use of BMQ among Jordanian adult patients with chronic diseases (Table 3). Data suitability for factor analysis was confirmed by KMO and Bartlett’s Sphericity Test results (0.85, p<0.001).

**Table 3:** Scores, factor loadings, communalities, corrected item total correlation, and Cronbach’s alpha if item deleted for BMQ items.

Statement	Mean score (SD)	Factor loading	Communalities	Corrected Item-Total Correlation	Cronbach’s Alpha if Item Deleted
**Factor 1**					
**My health, at present, depends on my medicine**	4.05 (0.94)	0.85	0.75	0.77	0.85
**My life would be impossible without my medicine**	3.75 (0.86)	0.84	0.73	0.75	0.85
**Without my medicine, I would be very sick**	3.58 (0.89)	0.88	0.77	0.79	0.85
**My health in future will depend on my medicine**	3.33 (0.76)	0.81	0.66	0.70	0.87
**My medicine protects me from becoming wore**	3.62 (0.84)	0.75	0.57	0.61	0.88
**Factor 2**					
**Having to take medicine worries me**	3.02 (0.90)	0.84	0.71	0.77	0.92
**I sometimes worry about the long-term effects of my medicine**	3.37 (0.98)	0.89	0.80	0.84	0.92
**My medicines are mystery to me**	3.24 (0.95)	0.91	0.82	0.86	0.91
**My medicines disrupt my life**	3.13 (1.10)	0.92	0.85	0.88	0.91
**I sometimes worry about becoming too dependent on my medicine**	3.32 (0.90)	0.84	0.71	0.77	0.92
**This medicine gives me unfavorable side effects**	3.59 (0.97)	0.78	0.61	0.70	0.93

**Fig 2:**
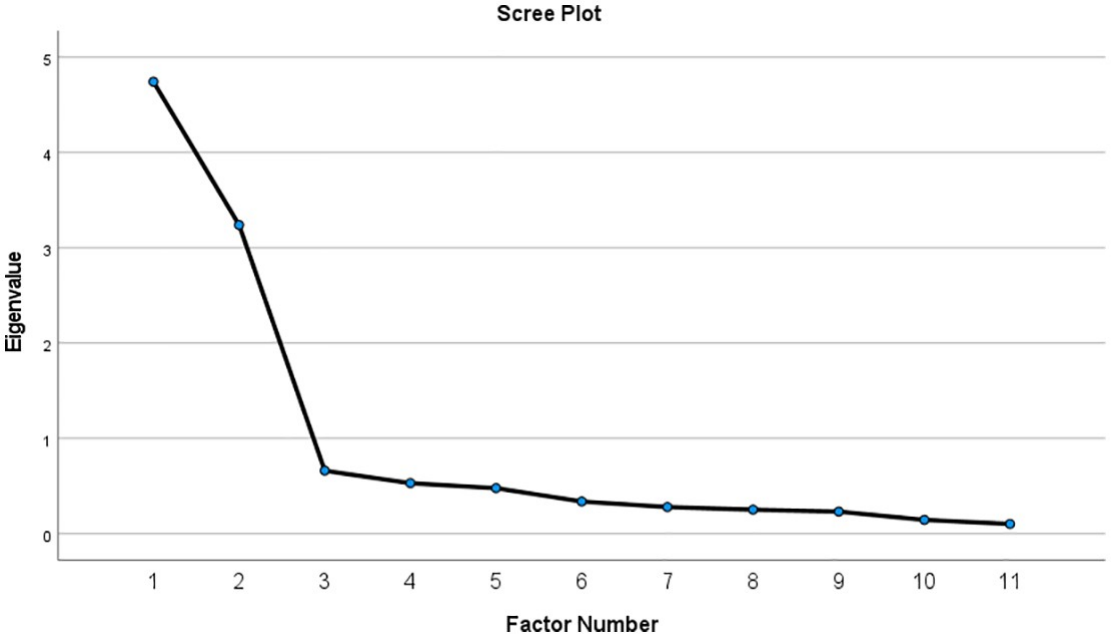
Scree plot of BMQ-specific.

Scree plot suggested two factors model (Fig 2) as there were two eigenvalue points above the line (elbow).

Internal consistency was confirmed by Cronbach’s alpha (0.89) for factor 1 and (0.93) for factor 2 and deleting any item did not enhance the consistency of the questionnaire’s items. The item with highest factor loading and communalities in factor 1 was “Without my medicine, I would be very sick”, while the item with the highest factor loading and communalities in factor 2 was “My medicines disrupt my life”.

#### Confirmatory factor analysis

**Fig 3:**
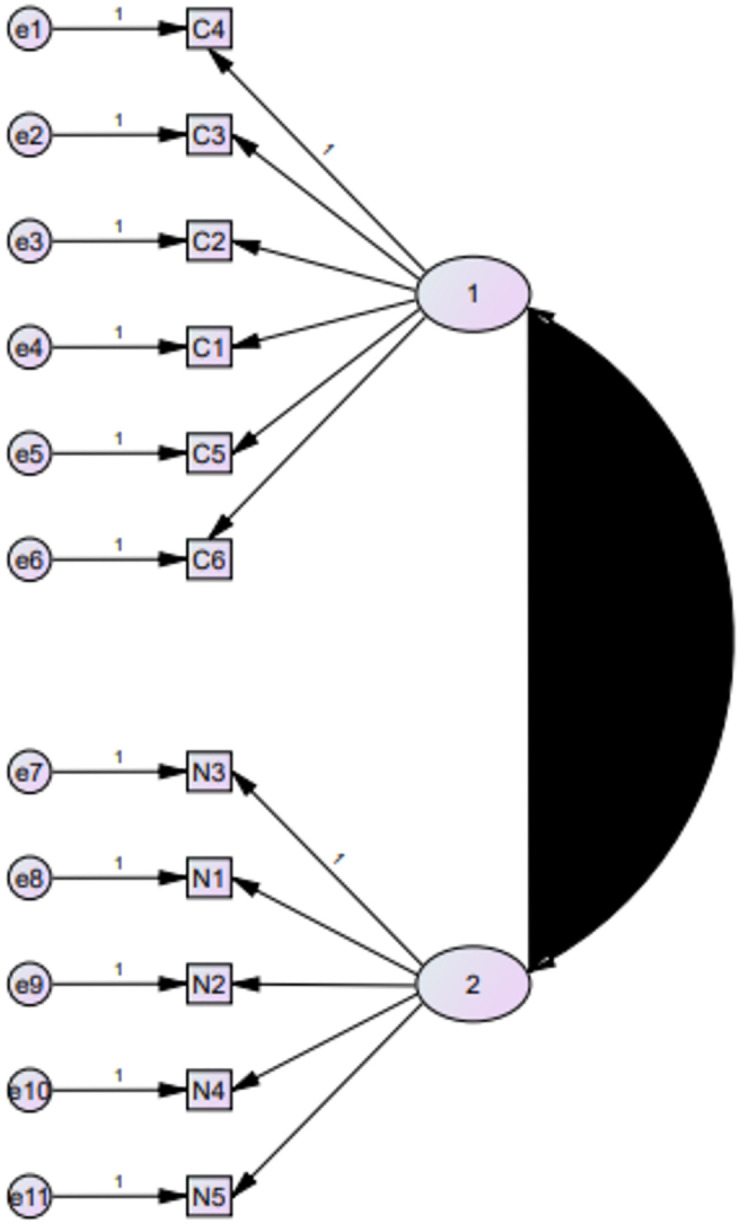
Confirmatory factor analysis for BMQ- specific.

CFA was conducted to evaluate the suitability of the models suggested by EFA for BMQ and MARS-5. The results of CFA confirmed the suitability for the two-factor model for BMQ (Fig 3) as indicated by different model fit indices including CFI = 0.97, CMIN/DF = 3.76, GFI = 0.57, SRMR = 0.04, and RMSEA = 0.07. Fig 3 illustrates the structure of the two-factor model of BMQ.

**Fig 4:**
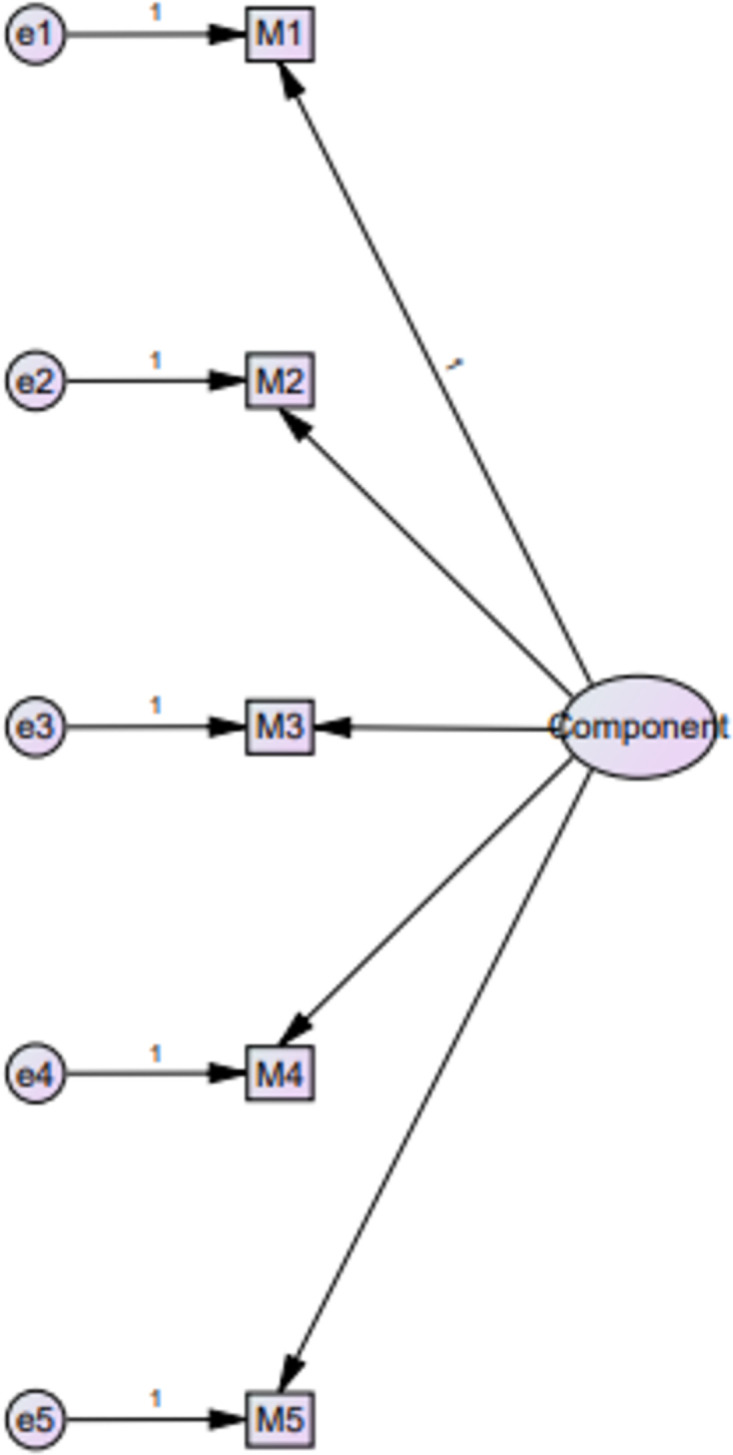
Confirmatory factor analysis for MARS-5.

CFA also confirmed the suitability for the one-factor model for MARS-5 (Fig 4) as shown in the following model fit indices CFI = 0.99, CMIN/DF = 2.62, GFI = 0.33, SRMR = 0.02, and RMSEA = 0.08. Fig 4 illustrates the structure of the one-factor model of MARS-5.

#### The ceiling and floor effects

The results indicated that only 45 patients (9%) scored the maximum possible score of 25 in MARS-5 and only 1 patient scored the least possible score of 5. In BMQ only one patient scored the maximum possible score in each factor. Only one patient scored the least possible score in the concerns factor and two patients in the necessity factor.

#### The convergent and discriminant validity of BMQ

As shown in Table 3, items in BMQ showed high loadings in the indicated factors. Furthermore, CR and AVE were in acceptable ranges for both concerns and necessity factors (0.95, 0.92 and 0.75 and 0.68 respectively). That is, the convergent validity was confirmed in this study (Table 4).

**Table 4:** Average Variance Extracted (AVE) and Composite Reliability (CR) for BMQ concerns and necessity factors.

Factors	CR	AVE	Square root of AVE	Latent constructs correlation
Concerns	0.95	0.75	0.87	***
Necessity	0.92	0.68	0.83	0.12

Items cross loading were evaluated and the highest cross loading was 0.173 for item ‘My life would be impossible without my medicine’, and the square roots of AVE for both factors were above the latent construct’s correlation (r = 0.12). Therefore, discriminant validity was confirmed.

## Discussion

Both BMQ-specific and MARS-5 questionnaires are well-validated tools in different clinical settings and different categories of patients [19, 35–37]. In the current study, BMQ-specific and MARS-5 questionnaires were furtherly validated on the studied sample to ensure the questionnaires were used properly and measure the intended outcomes reliably with minimum error or bias. Validation process should be conducted using representative number of samples. In this study, 485 recruited patients properly filled the questionnaires. This high number of filled questionnaires guaranteed a reasonable validation process and strongly indicated that both BMQ-specific and MARS-5 questionnaires are strong objective tools in terms of measuring their intended outcomes. The eigenvalues shown on the scree plot of MARS-5 advocated one factor model for MARS-5 and two-factor model for BMQ-specific. Cronbach’s Alpha is a measure of scale of reliability that demonstrates how closely related a set of items are as one group. Cronbach’s Alpha cut-off point is ≥0.7 [38] which is the case of the five items of MARS-5 (0.89) and 11 items of the two domains of BMQ-specific (0.89 and 0.93). Cronbach’s alpha scores when an item is excluded from the questionnaires were also reasonable for both MARS-5 (0.85–0.89) and BMQ-specific (0.85–0.88 and 0.91–0.93) for factors 1 and 2 respectively). The produced CR and AVE values for the two tools were acceptable and the factors loading in the intended factors were high indicating acceptable convergent validity of the BMQ instrument. In the BMQ two-factor model’s Items cross-loadings were low Fornell & Larcker criterion was met, indicating acceptable discriminant validity. This comprehensive validation and internal consistency suggest the suitability of employing both MARS-5 and BMQ-specific on the studied sample. Moreover, CFA confirmed the suitability of the two models.

Factor loading represents the correlation coefficient for the variable and factor, it shows the variance explained by the variable on that specific factor [39]. All factor-loading scores were higher than 0.7, item with the highest factor loading for MARS-5 was “I take medications less than instructed” and the mean score of this item was (3.49). These results illustrate the tendency of patients with chronic diseases generally to manipulate their recommended doses and take less medications or doses than instructed. On the other hand, “forgetting to take medications” was the lowest factor loading. This confirms to some extent that skipping doses in the studied sample was mostly intentional; driven by the beliefs about medications or diseases as illustrated by the mean score of the item “I decide to skip a dose” (3.51) and as described in the literature [40, 41].

The BMQ-specific factor 1 (necessity) with the highest loading was “without my medicines, I would be very sick” while the highest loading factor of concerns was “my medicines disrupts my life”. This finding indicates the fact that patients do develop strong beliefs about the chronic medications they take as they felt that their medications are very essential to maintain their health while at the same time, they believe that these medications influence their lives. The lowest loadings were “my medicines protect me from becoming worse” and “having to take medicine worries me” from necessity and concerns domains respectively.

The determinants of each BMQ factor imply that patients’ ideas about medication are coherent in "common-sense" terms. For example, patients were more likely to have strong beliefs in the necessity of their medication if it was perceived to affect their symptoms. In the current study, patients had strong positive attitude and belief that medications are important for good health, but also reported fears of consequences related to taking medications regularly like, side effects, duration of the use and dependence to medications. The BMQ-specific item with the highest necessity was “my health, at present, depends on my medicine” while the item “my health on the future will depend on my medicine” was the lowest reported in this study. In terms of the concerns, the item “this medicine gives me unfavorable side effects” was predominant, while “having to take medicine worries me” of the lowest apprehension.

Adherence to medications is widely studied in the literature. It was considered as an influential factor in reducing the rates of mortality and morbidity due to chronic diseases. Also, adequate adherence rate for a given condition is associated with reduced rates of hospitalization which could positively affect health care expenditure. A review study [42] included 19 studies, about medication adherence, showed that the rate of medication non-adherence in the Middle Eastern countries was estimated to be within the range of 1.4% to 88%. In our study, adherence rate was calculated to be 32%. Furthermore, factors identified to be influencing adherence to medications were necessity and concerns beliefs, dosage frequency and having medical [7].

Since 61.1% of the patients had high fears and concerns scores about long-term side effects of taking medications chronically, it is mandatory for pharmacists to be knowledgeable and fully aware of such fears. Pharmacists should also direct patients’ education and intervene to minimize such concerns and consequently minimize non-adherence. It is also for immense importance that pharmacists play their role in patient education as this is well known to improve the possible beliefs about medications in general, and consequently contributes to improve adherence to medications. For example, patients who take medications for chronic illness such as hypertension or diabetes mellitus need to know that their medications are not addictive and that medications have an acceptable safety profile for long-term use. Therefore, assessment of medication beliefs may be important for success of medication improvement strategies.

Patients’ concerns lead to hesitancy in taking medication, thus, patients’ education as a core of intervention is recommended to minimize the patients’ myth about their treatment plans. So, addressing causes behind non-adherence. Accordingly, the health care team can develop treatment strategies according to patients’ hopes and expectations, and allow patients to make decisions in therapeutic choice. This late conclusion imposes the concept of “concordance to medications “rather than “adherence to medications”. The former concept implies that both physician and patient discuss the treatment plan and the patient indirectly “promises” to adhere to such a discussed plan.

## Limitations

The present study is based on self-reported practices and beliefs which was not evaluated independently and might be subject to recall and social desirability biases. Furthermore, other health instrument validation techniques like concurrent validity and predictive validity were not applied in the current study due to the present study methodology.

## Conclusion

The Arabic versions of the BMQ and MARS-5 provide adequate tools to investigate patients’ beliefs about medication and medication adherence in Jordan and Arabic speaking countries, which can be used to evaluate and develop interventions to improve patients’ adherence and beliefs about medication.
